# Excerpta

**Published:** 1881-03

**Authors:** 


### EXCERPTA.

### DANGERS OF "BONWILL'S METHOD" OF ANÆSTHESIA.

This method, as is well known, is by means of forced respirations. Dr. Henry Gibbons, of San Francisco, says of it, in the *Pacific Medical Journal* :—

"We suppose that most persons, most boys, at any rate, have experienced the sensation produced by blowing at a fire, substituting their lungs for a bellows. The proposed method is nothing more than an exaggeration of this. So far from regarding it as likely to prove useful, we look upon it as decidedly dangerous. It puts the brain in an abnormal condition by interfering with the circulation, and is almost equivalent to partial strangulation. We are surprised that any considerate physician should recommend such a violation of physiological laws."

### INTERNATIONAL MEDICAL CONGRESS.

An international Medical Congress, with a Section for diseases of the Teeth, will be held in London, England, beginning August 2d and continuing till the 9th, inclusive, 1881.

On the general programme this constitutes Section 12.

The following list of subjects have been prepared for consideration at that meeting :

1st. Replantation and transplanting of teeth.

2d. Premature wasting of the alveoli, and its amenability to treatment.

3d. The share taken by septic agents in causing disease of the dental pulp and periosteum.

4th. Mercurial and syphilitic teeth, and the causes of irregularities of position of the teeth.

5th. New dental instruments and methods of operating.

Other topics may be introduced if time and opportunity will permit.

This will be an occasion of great interest and importance to the dental profession throughout the world. It is gratifying that in preparing and arranging for an international medical congress, dentistry has been assigned a position, and dentists invited to co-operate in the proceedings of the occasion.

A full and complete organization for this Section has been made by the profession in London, and of such a character as to insure success.

Mr. Edward Saunders, Esq., has been elected President of this Section, and Mr. C. S. Tomes, Esq., F. R. S., No. 37 Cavendish Square, W., Secretary.

The President and Secretary request all who intend to be present to notify them as early as possible, also to give any suggestions in regard to subjects for discussion.

A contribution of one guinea (or five dollars) will be required from those who are to be members, at the time of taking out their Cards of Admission. This will constitute membership, will admit to all the meetings, and will entitle each member to a copy of the Transactions of the Congress.

Such contributions may be sent to the President or Secretary. This, with notice of intention to be present, should be sent at an early day.—*Dental Register.*

### ABOUT COIN.

"Is there any law or statute forbidding the melting or working up the coin of the United States?"

In reply to this question, Section 5,459, Revised Statutes, says:—

"Every person who fraudulently, by any art, ways or means, defaces, mutilates, impairs, diminishes, falsifies, scales, or lightens the gold or silver coins which have been or may hereafter be coined at the mints of the United States, or any foreign gold or silver coins which are by law made current or are in actual use and circulation as money within the United States, shall be imprisoned not more than two years, and fined not more than two thousand dollars."

This, so far as I am aware, is the only law on this subject. It certainly does not forbid the melting of coin for the arts and manufactures.—*Dental Register.*

### THE SAD END OF A PROMISING LIFE.

The sad intelligence is just at hand, that, on Saturday, Jan. 29th,

Dr. W. F. Harbaugh, Dentist, of Piqua, Ohio, murdered his wife, and then killed himself, leaving three interesting children.

This terrible tragedy is the result of a career of dissipation from the excessive use of intoxicating drink, in which he had indulged for two or three years. During this time it had been recognized by his friends, and by all who knew him, indeed, that he was rapidly going the downward road to ruin; though none had anticipated so sad an end. Previous to contracting this terrible habit, Dr. H. was popular in the community in which he lived, and stood well in the profession, of which he had been for quite a number of years an honorable member.

He was a kind and affectionate husband and father when sober, but a very demon, it is said, when under the influence of liquor. A few short years ago none had a brighter prospect than Dr. H., and yet all was trampled in the dust at the behest of the accursed drink. The pleasure of those who indulge in the use of intoxicating drink is purchased at an awful cost, yet society seems determined to have it so.—*Dental Register.*

### HYPNOTISM, CATALEPSY AND THE FACULTY OF SPEECH.

It will not be at all amiss, in connection with the recently reported experiments in hypnotism of Prof Hammond, to mention a memoir by M. Ballet, which appeared in a recent number of *le Progres Medical.*

This memoir gives a curious means of demonstration, in the living subject, of the cerebral localization of the faculty of speech in the left hemisphere.

It is well known that for several years M. Charcot has plunged his hysterical patients at the Saltpetriere into the hypnotic or cataleptic, state as he chose, and by means as simple as those of Prof. Hammond.

These two conditions differ essentially; hypnotism is characterized by the following peculiarities: 1. When the limbs are elevated they do not remain in position, but fall when the support is withdrawn; the muscles and nerves are, however, in an excitable state, and irritation of any particular nerve induces contraction in the corresponding muscles with as much precision as the localized electrization of Duchenne of Boulogne. 2. The faculty of speech remains, the patient replies to questions, counts or recites verses, as he is commanded; he is even able to write or communicate with those about him by gestures. While in this hypnotized condition the patient may be, with facility, thrown into the cataleptic state. To bring this about it suffices to lift the eyelids and allow the light to influence the retina. The scene then changes completely; the hyper-excitability of nerves and muscles disappears; the limbs remain in whatever position they are placed; it is true catelepsy.

2d. What is yet more remarkable, every external intellectual manifestation is abolished; the patient no longer speaks or answers questions.

But what is even more astonishing, a patient may be placed in these two conditions at one and the same time; the right half being in a cataleptic state, while the left is in a hypnotic slumber, and *vice versa.* In order to bring this about the patient is hypnotized as usual, then one eyelid is raised while the other eye remains covered. Under these conditions the cerebral hemisphere which corresponds to the covered eye (on account of the crossing (entrecroisement) of the filaments of the optic nerve this is the opposite hemisphere) remains in a hypnotized state, while the hemisphere corresponding to the uncovered eye enters on a cataleptic condition.

Thus in this subject there exists at the same time a state of hemi-lethargy and hemi-catalepsy. M. Lepine originated, in Charcot's service, the experiment which we will briefly present, and which is at present daily repeated at the Saltpetriere. A hysterical patient is hypnotized; she speaks, reads, and writes while in this state; the left eyelid is raised, and the right hemisphere thus plunged into a cataleptic condition, and the patient continues to speak and gesticulate. But if, on the other hand, the right eye be uncovered, that is, if we place in the cataleptic state the left hemisphere, the presumed seat of the faculty of speech, the scene changes. The patient suddenly becomes silent, she no longer answers questions; the phrase commenced remains unfinished; the patient no longer makes sign or gesture.

A hysterical patient in the hypnotic state is commanded to count, and does so automatically. We suddenly uncover the left eye (right hemisphere), there is no interruption in the enumeration; we lift the right eyelid (left hemisphere); there is sudden arrest in the counting, but the patient recommences as soon as the eyelid is dropped.—*Medical and Surgical Reporter.*

Bolting Food.—There are some among us who think that the American habit of bolting indigestible foods and drinks—now hot and now cold—has done about as much to advance dentistry as anything.—*Johnstons' Dental Miscellany.*

Feminine Wisdom.—In the London Board schools recently a number of girls were examined on some physiological subjects. The answers contain some information new to American dentists. One said: "The loss of teeth is a serious matter, as we cannot *s-chew* our food enough." Another says: "Food has to be *jewed*, and *their* is a substance which helps to *jew* it, called saliva, and in that saliva *their* is a substance which is called ptyalin."—*Johnstons' Dental Miscellany.*

Longevity of Medical Men.—The calendars of the Royal College of Physicians and Surgeons, London, give some rare examples of longevity among their fellows and members, viz.: Archibald Billing has just completed his 90th year; Joseph Hurlock, of Brighton, is 88; Sir Thomas Watson, Bart., 88; Alexander Tweedie, 86; James Arthur Wilson, of Holmwood, 85; Bissett Hawkins, 84; Sir James Alderson, late President of the Royal College of Physicians, 80; Christopher J. R. Allatt, of Dover, 8[illegible]; Sir George Burrows, Bart., late President of the Royal College of Physicians, 79; James Muscroft, of Pontefract, 95; T. W. Greenhow, of Leeds, 90; Robert Tayler, of Brighton, 91; James Moncrieff Arnott, late President of the Royal College of Surgeons of England, 87; John Flint South, of Blackheath, 84; Cæser Henry Hawkins, Sergeant Surgeon to the Queen, 83; James Luke, Consulting Surgeon to the London Hospital, 83; Robert M'Cormick, R. N., Deputy Inspector of Hospitals and Fleets, 83. This gentleman accompanied Sir Edward Parry as assistant surgeon in Her Majesty's ship *Hecla*, in the attempt to reach the North Pole, in 1827.—*The College and Clinical Record.*

FOR ARSENIC POISONING, a very handy and efficient antidote can be made, by adding baking soda to dilute tinct. of Iron, forming sesquioxide of iron and common salt. This illustrates how readily ferric chloride adds its chlorine to another substance.—C. H. Shivers, M. D., *Haddonfield, N. J.*

IODINE AS A SPECIFIC IN CROUPOUS PNEUMONIA.—The treatment of croupous pneumonia has since some time lost its former active character; since we could but recognize that the course of the disease could not be influenced by any of the present plans of medication. A startling statement now appears by Dr. F. Schwarz, in the Deutsche medicinische Wochenschrift, (No. 2, 1881,) who claims that the disease may be aborted by iodine of iodide of potassium. He quotes statistics in the first place from the most reliable German sources, according to which the crisis occurred in 0.6 per cent. during the second day, in 4.7 per cent. during the third, in 7.4 per cent during the fourth, in 15.9 per cent. during the fifth, in 13.6 per cent. during the sixth, in 22.7 per cent. during the seventh, in 13 per cent. during the eighth and in 11.8 per cent. during the ninth day. These statistics refer to 933 cases of croupous pneumonia treated expectantly. In opposition to these figures Schwarz gives his results as the proof of the efficacy of his treatment. He has had altogether 98 cases, in ten of which the abortive treatment succeeded. This treatment is successful only when begun as soon as the disease starts. If instituted later it seems to be powerless. The inference to which the author leads us is that he saw none but the ten cases quoted at a sufficiently early period, but he does not state this point in so many words. By reproducing the temperature curves he proves that the aborted cases commenced in the usual characteristic manner. In all of these the crisis was completed during the second day. The defervesence took from 6 to 18 hours, on an average about 12 hours. The quantities given were very small, one sixth of a drop of tincture of iodine, or about one grain of the iodide being taken every hour. After the fever had ceased the local symptoms began to recede rapidly.—*Chicago Medical Review.*

TREATMENT OF PNEUMONIA.—Hiram Corson, M. D., in an article in the *Phila. Med. and Surg. Reporter*, concludes as follows:

You are anxious, by this time, to know how I regard this disease and how I would treat a case. To me, it is simply an inflammation, and I would use rapidly, as early as possible, the means which, in a practice of half a century, I have found safe and most efficient in allaying inflammation. Well, here is my patient; he had a chill hours ago ; he has some pain in his side, some cough, he feels very weak, his face is flushed, he is hot and thirsty, his pulse is over a hundred, the thermometer rises to 103°, he breathes scarcely at all through most one lung; there is a little blood and viscous fluid, slightly rust colored, in the basin by the bedside; he knows he got sick; he "was down in the iron ore shaft all yesterday, at work; came up in the evening to come home, and being in a perspiration, soon in facing the wind, cooled off too suddenly." I propose to bleed him ; he objects, on account of his great weakness, I tie up his arm, bleed him twenty ounces, or perhaps he feels faint by the loss of less than that; he expresses himself as being relieved somewhat of his oppression; he can lie more comfortably, he feels relieved of the fullness in the chest, but still he has a little pain in the side, or in the breast just above the nipple. I order him one-quarter gr. sulph. morph., direct that a towel wrung out of cold water, ice-water, if convenient, be applied over the affected part, to be changed frequently—or substituted by a bladder of ice and water. Then directing a mixture which contains some spirits of nitre, with about the 32d of a grain of sulph. morph. to a teaspoonful every two or three hours, I leave him, with an express direction not to worry him by any offer of food. Next day, or even the same day, perhaps, if I find that he is not entirely relieved of the pain, and is still expectorating viscid sputa, I bleed him again freely, and if need be, cup him, and after that he is generally safe. Some one will say—that may do very well for your strong iron-ore diggers, but it would prove fatal to our delicate patients. Perhaps so, but in my experience I have found that the weak and frail need to have the same means used to allay a toothache or a sciatica, a pleurisy or a rheumatism, as the robust. They bear the remedies to cure an ague as well, and need them as greatly, as the strongest. I have often bled the infant strangling with croup, with immediate relief, weak women with pleurisy or pneumonia, old men and aged, very aged women, to relieve affections of the brain, or avert a paralysis.

I believe there has not been any change in the human system in this climate which had made it more hazardous to take blood from the sick than it was forty years ago.—*Southern Practitioner.*

### NITRO-GLYCERIN IN ACUTE AND CHRONIC BRIGHT'S DISEASE, AND IN THE VASCULAR TENSION OF THE AGED.

Dr. A. W. Mayo Robson, (*Brit. Med. Jour.*, 1880, vol. ii. p. 803) has given nitro-glycerin in these cases with great benefit. A man of 56, with puffy eyelids and œdematous legs, a pulse tense and corded, the heart greatly hypertrophied, and breathing labored and difficult at times, was given a one-per-cent. solution of nitro-glycerin in one-minim doses every half-hour till its physiological effects were produced. It relieved the asthmatic symptoms so effectually that the patient would never afterwards be without it. After taking the medicine in three-minim doses thrice daily for a week, the urine, of which only a pint and a half daily, of specific gravity 1008, and very albuminous, had been passed, was now voided to the amount of three pints, specific gravity 1012, and almost free from albumen. This patient continued to take the medicine for some months, with great amelioration of the symptoms.

Dr. Robson mentions another similar case, in which the relief gained was equally striking. In the case of a woman of 52, who had had one slight apoplectic seizure and was threatened with another, and where the pulse was hard and corded and all her vessels indicated an increase of tension, nitro-glycerin was administered in one-minim doses thrice daily, with the result of removing entirely all symptoms of dizziness, etc. In the subsequent history of this patient, a dose of the remedy has been taken whenever dizziness has begun to come on, with the result of relieving the symptoms, and as may be supposed, of averting for the time a threatened attack of apoplexy.

A case of angina, or of anginaform attacks, appeared to be cured by the use of the nitro-glycerin. Other interesting cases are detailed by Dr. Robson, in which patients suffering an attack of acute nephritis were quickly relieved and cured.

Dr. Robson says, in conclusion, that whether the vascular tension which is the symptom treated be due to chronic kidney-mischief or to arterial fibrosis, this condition is unquestionable relieved by nitro-glycerin, and with the diminution of pressure, in his experience, improvement inevitably follows, though in some cases it may be only temporary.—*Philadelphia Medical Times.*

### ON THE PREVENTION OF LACERATION OF THE FEMALE PERINEUM.

Alex. Duke, M. K. Q. C. P. I., in the *Medical Press and Circular*, November 24:

The best authorities are, I think, agreed that it is not advisable to support the perineum when that important structure is distended by the passage of fetal head, and the reason is sometimes given that the support is so seldom properly applied that it is better left undone.

However, as it is a most deplorable accident to happen to any female, not only on account of the additional danger to the patient from sceptic absorption, the additional anxiety and trouble it gives to both nurse and doctor, and the train of subsequent evils which it frequently sets up, I consider it a subject worth saying a few words about, if only to draw out the opinion of older and wiser heads as to the advisability of adopting some preventive treatment instead of as a rule interfering at the wrong time, with the calamitous results we so often witness.

The best preventive treatment of laceration which I have found (and which I dare not claim as original, as I presume it has been tried before, but which I see no mention of in the text-books of midwifery) is this: When I find the head fairly engaged in the pelvis, and advancing with each pain, I take my seat by the patient's bed, and having lubricated my left thumb or the two first fingers on my right hand, I introduce either into the vagina, and at the outset of a pain draw back the perineum firmly, but gently, toward the coccyx, relaxing the tension as the pain lessens till the next ensues, and so on till I can draw back the perineum with very slight effort. I thus tire out the muscular structures, and produce sufficient relaxation for the head to pass. In most cases so treated the perineum is in no danger, but when the pubic arch is narrow, I take additional precaution to foment the parts, and use an inunction of lard, and also allow the head while passing through the valve to glide over my lubricating fingers, using them as a shoe-horn, so to speak, while I direct the head forward by pressure with my left hand below the coccyx or a finger in the rectum.

It has always seemed anomalous to me that the perineum should be expected to dilate on such short notice, namely, the "process of extension," (while dilatation of) the os and cervix occupy such a considerable time, even with the additional help of nature's hydrostatic dilator, viz.: the bag of waters.

The drawing back of the perineum produces no additional pain, as it is done during a uterine contraction, and I feel sure if nurses were educated as to the proper way of dilating the perineum previous to its distension with the fetal head, we should see and hear less of lacerated perineum.

High Potencies.—At the recent meeting of the New York State Homœopathic Society, Dr. H. M. Paine had the candor and boldness to say: "Our experience in the use of high potencies is based, as Hahnemann's was, on theoretical grounds only. It is one of the most singular forms of idealism ever seriously entertained by the medical profession. I firmly believe that when our reputed cures are reported in connection with all cases treated, we shall find that their frequency is not greater than those of daily occurrence, without medicine of any kind."—*Medical Record.*

### NEW METHOD FOR WITHDRAWING FLUIDS FROM THE MIDDLE EAR.

Dr. Charles Denison, of Denver, Col., states (in the Rocky Mountain Medical Review) that last winter, in the act of blowing his nose—having a cold at the time—catarrhal or other secretion was forced through the Eustachian tube into the middle ear, causing pain which was not relieved by the usual palliative measures. The experiment which gradually overcame the difficulty was this: Attaching the flat nozzle used with Pollitzer's air bag to the exhaust bottle of his aspirating apparatus, he first exhausted the air from the bottle, and then compressing the alæ of the nose, with the nozzle introduced so as to wholly exclude air from without, he closed his throat as in the act of swallowing, and kept it so, and at the same time directed the stop-cock to the vacuum bottle to be turned on. The effect of thus transferring the vacuum to the middle of the head was decided, seeming to draw everything inwards towards the pharynx and nasal passages, creating a temporary congestion of those parts. But the pain in the ear was perceptibly lessened by the experiment, and on its repetition finally wholly disappeared. He thinks that the effect was mechanical, and that a small portion of the fluid was probably drawn into the Eustachian tube at each operation. Since that time he has tried the experiment in practice as opportunity has offered, and mainly with results similar to those above mentioned. In one instance, he believes the stage of suppuration had set in, for he intended to puncture the membrana tympani the next day after this operation had been tried. But the patient did not return, aud he found some time afterwards that improvement had continued from that time till she wholly recovered.— *Va. Medical Monthly.*

### HOT RECTAL DOUCHE.

Dr. Chadwick, of Boston, in a paper before the American Gynecological Society, " Recommended the use of hot rectal douche for arresting diarrhœa and abdominal pain, and for pelvic inflammations of all kinds. Hot water thus brought into the abdominal cavity will encircle the whole mass of pelvic organs and produce a wonderful effect upon inflamed organs. After citing a number of cases, the Doctor remarked: " The water should be as hot as the hand can endure; while using the douche pass the finger into the vagina with the palm backward. The minute you begin to feel the lower pouch filling up you must pause a moment, without a withdrawal of the nozzle. In this wise from one to four pints of water may be introduced without exciting immediate action. The peristaltic action is very valuable. Sometimes it will be set up very soon, and sometimes not for hours. I am unable to say how far up the water usually passes, but I am satisfied it passes through the large intestine to the valve. Retrostalsis, I am satisfied, does not occur under some circumstances. I recommend its use two or three times per day for a week or so, and then sometimes to be discontinued for a week. I recommend the douche principally far an inflammatory condition of the large intestines or the rectum, secondarily, a condition of the pelvic organs characterized by painful defecation or burning sensations about the ovaries."—*Southern Medical Record.*

BERI-BERI.—Beri-beri is a constitution disease of an infectious nature, whose etiology is unknown; assuming a mixed condition of paralysis and œdema, characterized by dyspnœa; disorder of the organs of digestion, of respiration, of circulation, and principally of the nervous system. The disease properly belongs to the tropical regions, where it prevails endemically or epidemically.—*Southern Medical Record.*

FOR REMOVING NITRATE OF SILVER STAINS.—Ten parts sal ammoniac and ten parts of corrosive sublimate in one hundred parts of water, will remove silver stains from the hands and linen without damage.—*American Journal Pharmacy.*

The parish priest of Sendomi, in the diocese of Lerida, Spain, has declared that the last absolution, extreme unction and Christian burial will be refused to any parishioner who allows himself, or whose kindred allows him, to be treated by any but duly qualified medical practitioners. All who are treated homœopathically will be deprived of the rites of the Roman Catholic Church, and be treated as Moors or Jews.—*London Lancet.*

A CASE OF POLYURIA CURED BY ERGOT. is reported by Dr. E. McClellan, U. S. A. (*Louisville Medical News.*) The patient, a man aged thirty years, had had diabetes insipidus for many weeks, and passed from ten to twelve quarts of pale, acid urine daily. After quinine had been tried with no success, he was put upon ext. ergot, fl. ʒ ss., q. 4 h. In four weeks he was discharged cured.—*The Medical Record.*

HAIR DYE WITH ONE PREPARATION.—The following can be used for the wiskers as well as for the hair. None of them are, or can be patented :

Nitrate of silver.............................................1 ounce.
Distilled water..............................................8 ounces.
Water of ammonia, sufficient.

Dissolve the silver salt in water, and add ammonia to the solution until the precipitate formed at first is redissolved. This gives a black dye. To obtain a brown color, increase the proportion of ammonia and water.

Bismuth Hair Dye.—

No. 1.

Citrate of bismuth..........................................1 ounce.
Rose water.......................................................2 ounces.
Distilled water...............................................2 "
Alcohol............................................................5 drachms.
Ammonia, sufficient.

No. 2.

Hydrosulphate of soda...................................12 drachms,
Distilled water...............................................4 ounces.

Each solution is to be applied separately, the No. 1 first, and No. 2 when this has dried thoroughly. To insure success, the hair must previously be well freed from all oily substances, natural or added, by means of some of the weak alkaline solutions commonly known as shampoos. The hair, being then rapidly dried, is ready to receive the dye. These remarks apply to all hair colorings.

—*Drug. Cir.*

### CORNS.—HOW TO REMOVE THEM.

Saturate a small piece of cotton with alcohol, apply it to the corn for a minute, then with a sharp scalpel or knife carefully separate the corn from the healthy tissues, which is easily done by a careful handling of the knife and gentle pulling with forceps, while the parts are being immersed with alcohol. If the alcohol dries away while operating, apply the saturated cotton again, and I frequently find it necessary to apply this several times before the operaton is completed. The alcohol not only lessens the sensibility of the parts, but it facilitates the separation of the hard corn from the soft and tender tissues. *This cures,* and that without drawing a drop of blood, or producing any pain, except what results from pulling on the corn with the forceps. After raising one edge, it is about like removing a piece of adhesive plaster.—*American Medical Journal.*

### THERAPEUTICS OF NITRO-GLYCERINE.

Various experiments have proven nitro-glycerine to be a powerful medicinal agent, and one which promises to be of much value in certain diseases. It has been suggested as a remedy for tetanus, hydrophobia, chloroform poisoning, and many other morbid conditions; particularly those of the nervous system. It is especially serviceable in neuralgia, having in many instances given relief when other remedies of recognized value have failed. In angina pectoris, a most distressing and often unmanageable affection, it has been most useful, and in many cases has effected a complete cure.

In over-doses its action is often unpleasant and dangerous, causing faintness and nausea, headache, fullness in the head, ringing in the ears, pulsation all over the body, unconsciousness accompanied by sterterous breathing and convulsive movements of the muscles of the face.

Its action is rapid, and seems to commence the moment it is swallowed. It is difficult to lay down any absolute rule as to dosage. It may be used in solution either with alcohol or ether, the former being preferable, and in strength of one per cent. The dose of this may vary from one to five drops, according to the susceptibility of the patient and the peculiarities of the case; sometimes even one drop is too large a dose. The repetition of the dose will also vary with the urgency of the symptoms from one to four hours.

Melted cocoa butter has also been used as a menstruum, as the nitro-glycerine is soluble in it, and the mass may be employed as a basis for making pills. The pills, however, do not act as quickly as the alcoholic solution, and it has been suggested to add an equal quantity of chocolate paste to the mass and form it into lozenges, which may be dissolved in the mouth before being swallowed.

In solution or mass nitro-glycerine can be dispensed and used in medicine with perfect and absolute safety. With the most ordinary care it would be impossible for an accident to occur.—*Abstract from British Med. Journal.*

### JANU'RY REPORT OF DEATHS AND INTERMENTS IN BALTIMORE.

| CAUSE OF DEATH. | No. | CAUSE OF DEATH. | No. | CAUSE OF DEATH. | No. |
|---|---|---|---|---|---|
| Abscess | 4 | Drowned | 1 | Leucocythermia | 1 |
| Abortion | 3 | Dysentery | 2 | Myelitis | 2 |
| Adenitis | 2 | Endocarditis | 1 | Malformation | 2 |
| Angina Pectoris | 1 | Epilepsy | 3 | Mal-nutrition | 2 |
| Apoplexy | 13 | Emphysema | 1 | Marasmus | 11 |
| Anthrax | 1 | Erysipelas | 4 | Measles | 1 |
| Ascites | 1 | Effusion of brain | 1 | Meningitis | 9 |
| Asphyxia | 2 | Endo peri metritis | 1 | " Tubercular | 9 |
| Asthma | 3 | Exposure | 2 | " cerebro spinal | 3 |
| Asthenia | 3 | Entero Colitis | 3 | " basilar | 1 |
| Actelectasis Pulmonum | 2 | Fever, malarial | 1 | Murder | 1 |
| Bright's Disease | 8 | " puerperal | 1 | Neuralgia | 1 |
| Burns | 4 | " scarlet | 58 | Old Age | 25 |
| Bronchial catarrh | 1 | " typhoid | 9 | Oedema of lungs | 1 |
| Catarrh of stomach | 1 | " typho-malarial | 1 | Paralysis | 13 |
| " lungs | 1 | " catarrhal | 3 | Pneumonia | 73 |
| Capillary Bronchitis | 11 | " intermittent | 2 | " typhoid | 5 |
| Cancer, Diffused | 2 | " gastro enteria | 1 | " broncho | 5 |
| " Breast | 2 | Fracture, rib | 1 | Parotitis | 1 |
| " Cervix | 1 | " skull | 2 | Premature birth | 7 |
| " Liver | 1 | Gangrene, navel | 1 | Phthisis, mucosa | 1 |
| " Uterus | 1 | " senile | 2 | Protracted labor | 1 |
| " Stomach | 4 | Gastro enteritis | 1 | Pyæmia | 2 |
| Calculus, bilious | 1 | Gout | 1 | Rheumatism, inflammatory | 2 |
| Cerebral Thrombosis | 1 | Hernia, inguinal | 1 | Scrofula | 6 |
| Congestive Chill | 2 | Hemorrhage, stomach | 2 | Softening of brain | 1 |
| Cirrhosis of Liver | 2 | " lungs | 1 | Spasm of heart | 1 |
| Concussion of Brain | 1 | " post partum | 1 | Septicæmia | 3 |
| Congestion of Brain | 8 | " uterus | 1 | Stricture œsophagus | 1 |
| " Lungs | 5 | " umbilicus | 2 | Starvation | 1 |
| " General | 1 | " brain | 3 | Scalds | 2 |
| Consumption of Lungs | 126 | Hydrocephalus | 3 | Suicide | 2 |
| " Bowels | 1 | Inanition | 10 | Syphilis | 3 |
| Convulsions | 36 | Inflammation of bladder | 1 | Tabes Mesenterica | 2 |
| Croup | 20 | " bowels | 2 | Tetanus | 3 |
| Charet | 1 | " stomach | 2 | Tumor, mammary | 1 |
| Cyanosis | 2 | " bronchi | 18 | " ovarian | 1 |
| Debility | 1 | " kidneys | 5 | Uræmia | 2 |
| Dentition | 6 | " larynx | 1 | Unknown, Adult | 1 |
| Dilatation of heart | 1 | " liver | 3 | Unknown Infantile | 4 |
| Diarrhœa | 3 | " pericard'm. | 1 | Varicella | 1 |
| Diphtheria | 47 | " peritoneum | 3 | Whooping Cough | 5 |
| Disease of heart | 22 | " pleura | 1 | Wounds | 5 |
| " brain | 1 | Intussusception | 1 | | |
| " spine | 3 | Intemperance | 3 | Total | 762 |
| Dropsy, (Gen'l) | 6 | Jaundice | 1 | | |

THE DEATHS REGISTERED IN THE MONTH INCLUDE

| | | | |
|---|---|---|---|
| Under 1 year | 172 | Between 20 and 30 " | 72 |
| Between 1 and 2 years | 53 | " 30 and 40 " | 51 |
| " 2 and 5 years | 79 | " 40 and 50 " | 44 |
| | | " 50 and 60 " | 47 |
| Under 5 years | 304 | " 60 and 70 " | 68 |
| Between 5 and 10 years | 59 | " 70 and 80 " | 41 |
| " 10 and 15 " | 24 | " 80 and 90 " | 21 |
| " 15 and 20 " | 26 | " 90 and 100 " | 5 |

NATIVITY, ETC.

| | |
|---|---|
| United States—White | 484 |
| " Colored | 168 |
| Foreign | 110 |
| Total for the month | 762 |
| Still Birth | 68 |

Among the decedents were 61 above the age of 70 years, whose combined ages were 4,809 years, averaging 78 years 10 months.

Estimated Population, 393,796. white, 338,384. Colored, 55,412. Annual Death Rate during the month, whole population, per 1,000—23.20. White, 21.08. Colored, 36.65.

Births reported, 824.

By Order: JAMES A. STEUART, M. D., Commissioner of Health and Registrar.

A. R. CARTER, Secretary.

U. S. SIGNAL OFFICE, BALTIMORE, MD., February 1, 1881.

### METEOROLOGICAL SUMMARY FOR THE MONTH OF JANUARY, 1881.

#### PRESSURE.

Mean Monthly Barometer, - - - - - 30.183 inches.
" " " for past 10 years, - - 30.170 "
Highest Barometer, - - - - - - 30.720 " on the 18th.
Lowest " - - - - - - - 29.223 " on the 21st.
Monthly Range of Barometer, - - - - - 1.487 "

#### TEMPERATURE.

Mean Monthly Thermometer, - - - - - 30.8 degrees.
" " " for past 10 years, - 35.9 "
Highest Thermometer, - 45 degrees on 13th. Monthly Range, - 51 degrees.
Lowest " - - 6 " below zero on 1st. Mean Daily Range, 12.3 "

#### WIND, ETC.

Prevailing Direction of wind, West. Total Amount of Rainfall, 4.84 inches.
Mean Velocity of Wind, 6.2 miles per hour. Number of days on which Rain fell, 14.
Highest Velocity of Wind, 19 " " " on 21st. Mean Relative Humidity, 71.6 per cent.
Total Movement of Wind, 3.673 miles ROBERT SEYBOTH, in charge

